# Mesenteric Ischemia and Myocardial Infarction Associated with Atrial Fibrillation

**DOI:** 10.1155/2018/7860397

**Published:** 2018-04-01

**Authors:** Liting Cheng, Yongquan Wu

**Affiliations:** Cardiovascular Center, Beijing Friendship Hospital, Capital Medical University, 95 Yong'an Road, Xicheng District, Beijing, China

## Abstract

Atrial fibrillation is a common disease correlated with embolism incidents. However, there is lack of report on atrial fibrillation causing myocardial infarction and mesenteric ischemia at the same time. Our patient is a 69-year-old woman who had undergone thoracic surgery a month before presented to our hospital with newly discovered atrial fibrillation, abdominal pain, and ST-elevated myocardial infarction. This is a rare case that atrial fibrillation took place one month after surgery and caused embolism incidents in both coronary artery and mesenteric artery.

## 1. Introduction

Recently, the relationship between stroke and atrial fibrillation has been well studied by both cardiologists and neurologists. However, there is lack of report on atrial fibrillation causing myocardial infarction and mesenteric ischemia at the same time. Here, we present a case in which atrial fibrillation was related to thoracic surgery and finally caused myocardial infarction and mesenteric ischemia simultaneously.

## 2. Case Presentation

**Figure 1:**
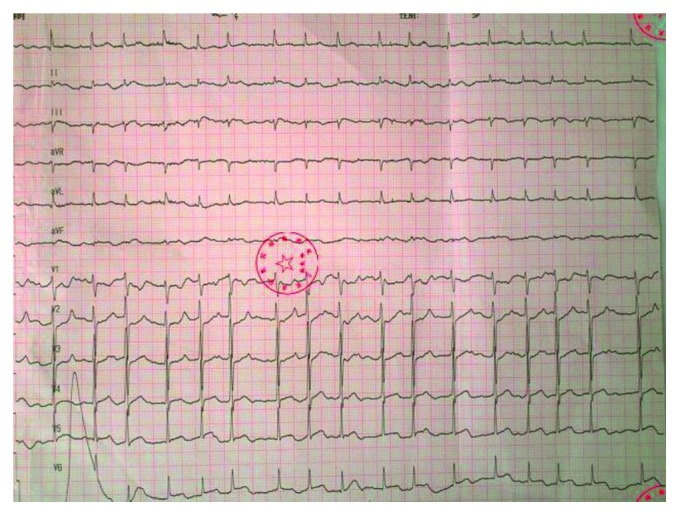
Electrocardiogram preformed on the emergency department.

A 69-year-old woman who had undergone surgery for left lung adenocarcinoma 1 month ago presented to the hospital with 8 hours of abdominal pain and 6 hours of palpitation. The physical examination revealed that her pulse was 68 beats per minute and the respiratory rate was 20 breaths per minute; her heart rate was 107 beats per minute with an irregular first heart sound three thoracoscopic surgical wounds could be found in the left chest wall bowel sounds were normal and there was no diffuse abdominal tenderness. The laboratory tests were as follows: granulocyte count, 82.8 (1.8–6.3) × 10^9^ per liter; fibrin degradation products level, 24 (0–5) milligram per liter; D-dimer level, 7.9 (0–1.5) milligram per liter; creatine kinase-MB, 6.9 (0–6.6) picogram per millimeter; troponin I, 3.153 (0–6.6) nanogram per millimeter; troponin T, 0.32 (0.010–0.017) nanogram per millimeter. Her previous Holter result showed no sign of atrial fibrillation, while the 12-lead electrocardiogram at the emergency department showed atrial fibrillation with ST-segment elevations (1 to 2 mm) in II, III, aVF, and V5–V6 (Figure 1).

**Figure 2:**
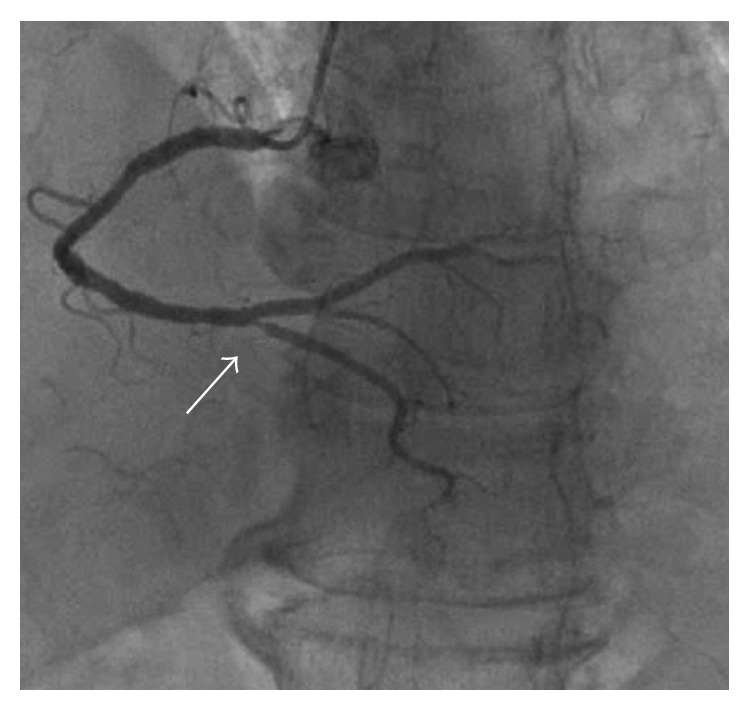
Right coronary artery. A thrombotic occlusion could be found in the posterior lateral descending artery.

The patient had a history of prolonged ongoing (greater than 20 minutes) rest pain that is less than 12 hours in duration; cardiac troponin T, troponin I, and creatine kinase-MB were elevated; the ECG indicated ST-segment elevated myocardial infarction. Urgent coronary angiography was preformed which identified no crucial stenosis in the left anterior descending artery, left circumflex artery, and posterior descending artery. However, a thrombotic occlusion could be found in the posterior lateral descending artery (Figure 2). No intervention was given to the patient.

**Figure 3:**
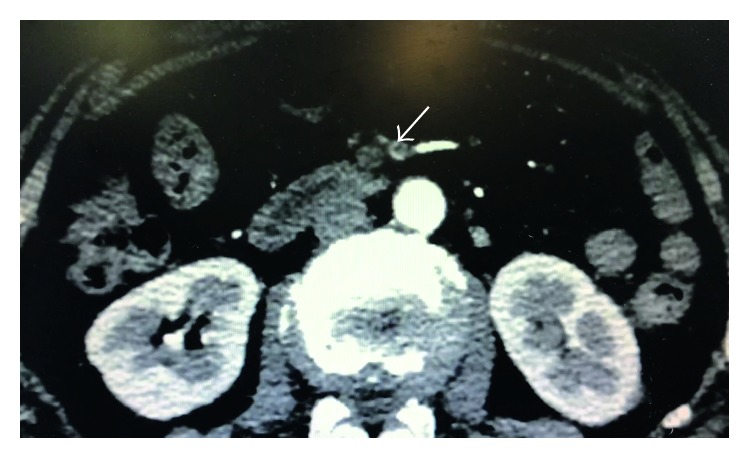
Abdominal computed tomography with contrast material showed occlusion of the superior mesenteric artery.

A treatment combined aspirin, clopidogrel, low-molecular-weight heparin, angiotensin-converting enzyme inhibitors, metoprolol, and pantoprazole was prescribed to our patient. However, she was stopped giving aspirin and low-molecular-weight heparin because the occult blood test turned positive and the platelet account started to decrease. The abdominal pain diminished after the angiogram; however, among the next 5 days, the abdominal discomfort occurred again and again without any causes and aggravated by eating. Abdominal computed tomography with contrast material showed occlusion of the superior mesenteric artery (Figure 3).

Considering that the mesenteric ischemia was caused by the embolism, rivaroxaban and clopidogrel were given to the patient. The vital of this patient is well during the follow-up.

## 3. Discussion

Atrial fibrillation, known as the most common arrhythmia, is highly related with embolism incidents. It has been reported that more than 3% of patients with ischaemic stroke in the South Asia and almost 20% in Western Europe, North America, and Australia are associated with atrial fibrillation [1]. Furthermore, the embolization of the peripheral extremities and arteries (splenic artery, renal artery, and mesenteric artery) are also correlated with atrial fibrillation [2]. However, both myocardial infarction and mesenteric ischemia attributed to atrial fibrillation are lack of report. Our patient presented to the emergency department with both abdominal pain and chest pain. Due to the elevation of cardiac troponin T, troponin I, creatine kinase-MB, and ST-segment in electrocardiogram, an emergency angiogram was performed. The occlusion of the posterior lateral descending artery was found which explains the myocardial infarction. Abdominal computed tomography revealed occlusion of the superior mesenteric artery.

The auxiliary examination, angiogram along with abdominal computed tomography, indicated it was a patient with both coronary and superior mesenteric artery embolism.

But what did cause atrial fibrillation? The risk factors of atrial fibrillation are variable which include valvular heart disease, cardiomyopathy, congenital heart disease, thyroid dysfunction, and so on [3]. On the contrary, the patient had no history of any kind of the diseases mentioned above. The Holter performed right before the thoracic surgery showed no sign of atrial fibrillation. Could the paroxysmal atrial fibrillation correlate with the surgery? Postoperative atrial fibrillation caught the eyes of doctors most recent years. It has been reported that more than 10% of patients who underwent anatomic lung resection had postoperative atrial fibrillation [4]. However, the importance of rhythm assessment after surgery is still short of knowledge. The CHA2DS2-VAS score of our patient was up to 4 (hypertension, diabetes mellitus, age, and sex), in which according to the previous study anticoagulation was needed [5, 6]. Unfortunately, no rhythm assessment was given to the patient, and anticoagulants were not administered which finally led to myocardial infarction and mesenteric ischemia. Therefore, it is significant to give patients with previous thoracic operation rhythm assessment to prevent further embolism incidents.
